# Investigation of comorbid autoimmune diseases in women with autoimmune bullous diseases: An interplay of autoimmunity and practical implications

**DOI:** 10.1097/JW9.0000000000000053

**Published:** 2022-10-07

**Authors:** Meropi Karakioulaki, Dedee F. Murrell, Aikaterini Kyriakou, Aikaterini Patsatsi

**Affiliations:** a Second Department of Dermatology, School of Medicine, Aristotle University, Papageorgiou Hospital, Thessaloniki, Greece; b Department of Dermatology, St. George Hospital, Faculty of Medicine, University of New South Wales, Sydney, Australia

**Keywords:** autoimmunity, bullous diseases, pemphigoid, pemphigus, women

## Abstract

Autoimmune bullous diseases are a group of skin disorders resulting from an autoimmune reaction against intercellular adhesion molecules or components of the basement membrane of skin and mucosa. Autoimmune disorders often occur in patients with a history of another autoimmune disease and most autoimmune diseases have a striking female predominance. In this review, we aim to analyze the different associations of autoimmune bullous diseases with other autoimmune diseases and highlight the distinctiveness of the female gender in these associations.

**What is known about this subject in regard to women and their families?**

- Autoimmune bullous diseases (AIBD) are a group of skin disorders resulting from an autoimmune reaction against intercellular adhesion molecules or components of the basement membrane of skin and mucosa and are often associated with high degree of morbidity (eg, pain, pruritus, impaired activities of daily life) and occasional mortality. AIBD have a significant female predominance and present many associations with other severe autoimmune diseases. In the majority of AIBD, there is an overall female predominance. Moreover, the quality of life of female patients with AIBD is affected more than men, mainly due to factors such as time spent on treatment, social misconceptions, physical discomfort, and financial burden. Additionally, pregnancy and lactation both raise some complicated management issues, including some treatment limitations and a fluctuating disease course with postpartum exacerbations or relapses.

**What is new from this article as messages for women and their families?**

- In this review, we aim to analyze the interplay of AIBD with autoimmunity, highlighting the distinctiveness of the female gender. Clinicians should be aware of these associations, especially when treating women with bullous diseases, in order to be able to aid them with serious decisions regarding pregnancy and lactation planning.

## Autoimmune bullous diseases

Autoimmune bullous diseases (AIBD) are a group of skin disorders resulting from an autoimmune reaction against intercellular adhesion molecules or components of the basal membrane (BM) of the skin and mucosal surfaces and are often associated with high degree of morbidity and occasional mortality.^1^

More specifically, pemphigus encompasses a group of autoimmune intraepidermal blistering diseases, divided into 2 major categories: pemphigus vulgaris (PV) and pemphigus foliaceus.^2^ It is mainly mediated by IgG autoantibodies directed against structural proteins of the desmosomes at cell–cell junctions, such as desmogleins (Dsg)-1 and 3 and other cadherin-type cell–cell adhesion molecules.^3–7^ This results in intraepidermal blister formation in the skin and mucous membranes due to keratinocyte detachment and acantholysis.^8^ Pemphigus shows an annual incidence rate between 0.1 and 0.5 per 100,000 people and has been reported more frequently in females, with a female to male ratio between 0.45 and 5.^2,9^ It may occur in all age groups, but the disease is most frequently diagnosed between the ages of 40 and 60.^2^

The pemphigoid group represents a group of autoimmune disorders characterized by subepidermal blistering, including the following forms: bullous pemphigoid (BP), dermatitis herpetiformis (DH), and epidermolysis bullosa acquisita (EBA).^2^ It is mediated by IgG autoantibodies against structural proteins of the hemidesmosomes at the dermal–epidermal junction, such as BP180, BP230, laminin 332, α6β4 integrin, and type VII collagen (C7), that lead to the formation of tense blisters and erosions on the skin or mucous membranes.^10^ BP is characterized by the development of IgG autoantibodies directed against 2 different components of the hemidesmosomes: Bullous Pemphigoid Antigen 1 (BP230 or dystonin) and Bullous Pemphigoid Antigen 2 (BP180 or type XVII collagen).^11,12^ Binding of these autoantibodies leads to complement activation, mast cell degranulation, and accumulation of inflammatory cells that release proteases, leading to cleavage of the BM and blister formation.^12^ It is manifested clinically by pruritic urticarial papules and plaques, forming vesicles and tense subepidermal bullae on an urticarial, erythematous base as they progress.^13^ DH is an autoimmune skin blistering disease characterized by IgA deposits in prilesional skin and it is the cutaneous manifestation of celiac disease.^14^ Epidermal transglutaminase (TG3) is the autoantigen which causes IgA deposition in the skin and tissue transglutaminase (TG2) is the autoantigen which leads to IgA accumulation in the small bowel mucosa.^15^ EBA is another rare (0.17-0.26 new cases per million people per year) AIBD that is characterized by IgG and IgA antibodies directed against C7, which is a major component of the fibrils that anchor the BM to the dermis.^16–18^ As a result, patients are typically affected by blisters, erosions, scars, milia, and nail loss.^19–21^

**Fig. 1.**
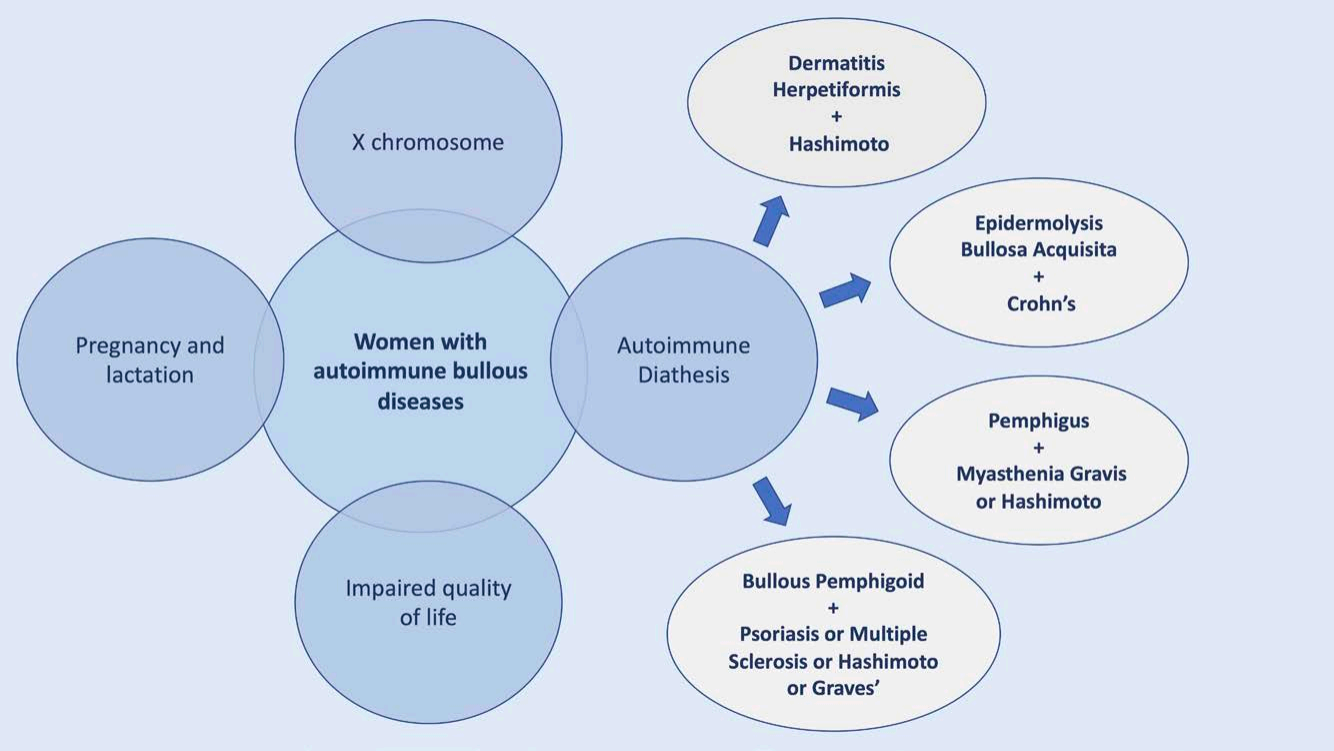
Predisposition, implications and autoimmune diathesis in women with autoimmune bullous diseases.

Autoimmune disorders occur in 25% of patients with a history of another autoimmune disease.^16,22^ This concept is known as autoimmune diathesis (Fig. 1) and indicates the need for continuous surveillance for the development of a new autoimmune disease in predisposed patients (Table 1).^16,23^

**Table 1:** Possible pathophysiological mechanisms leading to significant associations between AIBD and other autoimmune diseases

AIBD	Autoimmune disease	Pathophysiological mechanism
BP	Psoriasis	Psoriatic degradation of laminin 1 and laminin a1 in the BM lowers the threshold for the generation of anti-BM autoantibodies that are also involved in BP^24^
		Neutrophilic infiltrate histologically present in both conditions.^25^ Neutrophils secrete several metalloproteases that may be implicated in the degradation of matrix proteins, leading to the exposure of antigenic epitopes of the BM^26^
		IL-1 is essential for the initiation and formation of psoriatic lesions^27^ and is also increased in blister fluid of BP^28,29^
		IL-17 and T helper type 17 cells play a major role in the pathogenesis of both BP and psoriasis^30,31^
		The breakdown of the BM by antipsoriatic treatments may facilitate the exposure of BM antigens to the circulation and generate anti-BP autoantibodies^32–34^
BP	Neurological diseases	Co-expression of epithelial and neuronal isoforms of BP autoantigens (BP180 and BP230) in the skin and the central nervous system^35^
		Autoantibodies against the neuronal isoforms of BP180 and BP230 may lead to neuroinflammation and may expose these antigens to the immune system resulting in a cross-reactive immune response against their cutaneous isoforms, leading to BP^36^
Pemphigus	Myasthenia gravis	Both pemphigus and MG are mediated by antigen-specific autoantibodies of the IgG4 subclass^5,37^ and are associated with similar variants of the HLA class II region, such as DRB1*14, DQB1*05, and DRB1*14^38^
		MG autoantibodies against nAChR are also reported in pemphigus,^39^ because mAChR and nAChR are both expressed on the keratinocyte membrane and regulate cell adhesion synergistically^40^
Bullous skin diseases	AITD	Keratinocytes, melanocytes, and dermal fibroblasts have shown to bear functional TSHR and other thyroid-specific antigens, such as Tg, TPO, and NIS^41–45^
		The expression of TSH has been reported in the normal human epidermis and it was shown to be up-regulated by TRH and down-regulated by thyroid hormones^41^
		TSH treatment of human skin in vitro has shown to increase the expression of involucrin, loricrin, keratin 5 and 14 to stimulate human keratinocyte proliferation^41,44^
Pemphigus vulgaris	Hashimoto thyroiditis	The absence of HLA type DQB1*05:03 predisposes a patient with PV to develop anti-TPO antibodies^46^
		The absence of both anti-Dsg 1 and anti-Dsg 3 autoantibodies in PV has been correlated with increased anti-TPO and anti-Tg^46^
EBA	Crohn’s disease	Expression of C7 in both colon and skin leads to a cross-reaction to the same antigen in different tissues^12^
		In EBA patients, C7 could harbor the primary epitope of a spreading phenomenon toward IBD autoimmunity^21^
		C7 expressed in the mucosa of the colon is altered by chronic inflammation of IBD and thus cryptic epitopes of the protein are revealed, or neo-epitopes are generated.^47,48^
		Molecular mimicry: the immune response directed against pathogens or the intestinal flora could induce the initial activation of T and B cells, which are cross-reactive with C7 epitopes provoking the generation of autoantibodies that trigger blister formation in the skin^47^

AIBD, autoimmune bullous diseases; AITD, autoimmune thyroid diseases; BP, bullous pemphighoid; BM, basal membrane; C7, collagen VII; Dsg, desmoglein; EBA, epidermolysis bullosa acquisita; IBD, inflammatory bowel disease; IL, interleukin; mAChR, muscarinic acetylocholine receptor; MG, myasthenia gravis; nAChR, nicotinic acetylocholine receptor; NIS, natrium/iodide symporter; PV, pemohigus vulgaris; Tg, thyroglobulin; TPO, thyroperoxidase; TRH, thyrotropin-releasing hormone; TSH, thyroid-stimulating hormone; TSHR, thyrotropin receptor.

## Autoimmune diseases and the female gender

Most autoimmune diseases have a striking female predominance with a female: male ratio as high as 10:1(Fig. 1).^49–51^ Interestingly, the X-chromosome contains the largest number of immune-related genes in the whole human genome.^52^ Females are mosaics for the X-chromosome, due to the inactivation of the X-chromosome, an epigenetic process that takes place early during the female embryonic development.^52^ It mainly refers to a random packaging of alleles into transcriptional inactive heterochromatin, irrespectively of the paternal origin of the X-chromosome.^52^

Most X-linked primary immune deficiencies demonstrate significant autoimmune manifestations, indicating the importance of the X-chromosome genes in the onset, function, and homeostasis of the immune system.^53–56^ Males seem to be less likely to develop autoimmune diseases as they carry only the maternal X-chromosome and their immune system has to cope with 1 set of proteins generated by the X-linked genes. Females, on the other hand, carry both the maternal and the paternal X-chromosomes, hence despite random inactivation causing only 1 copy per cell, in the systemic circulation there are proteins which have slight variations due to these polymorphisms. This may have a damaging effect on the immune system homeostasis, as autoreactive immune cells cannot tolerate self-antigens encoded by 1 of the 2 X-chromosomes and therefore, they trigger autoimmune responses in target tissues.^49,52^

In the majority of AIBD, including pemphigus, pemphigoid, and EBA, there is an overall female predominance.^13^ Moreover, the quality of life of female patients with AIBD is affected more than men, mainly due to factors such as time spent on treatment, social misconceptions, physical discomfort, and financial burden.^13,57,58^

Additionally, pregnancy and lactation both raise some complicated management issues, including some treatment limitations and a fluctuating disease course with postpartum exacerbations or relapses.^13^ Indeed, hormonal changes during pregnancy may potentiate autoimmune disease activity, especially when the disease is uncontrolled at the time of fertilization.^59^ Moreover, during pregnancy, women are highly susceptible to autoimmune diseases, due to the necessarily immunotolerant state that occurs during childbearing.^60^ Some AIBD, such as pemphigoid gestationis present their initial onset during pregnancy, while others may improve or flare during pregnancy or postpartum.^13^ PV improves after the third trimester of pregnancy and then flares postpartum,^61^ while BP is very rare during pregnancy and mainly affects the elderly.^13^ For pregnant women with AIBD, the delivery should be vaginal, as slow wound healing may occur after cesarean delivery, associated with corticosteroid use.^62^

Autoimmune diseases in females usually occur after puberty and sexual activity.^63^ After unprotected sexual intercourse, male sperm may be found into the peritoneal cavity of women exposing the immune system to foreign proteins.^64^ Additionally, within the cervix, seminal fluid interacts with female tissues and induces inflammation-like responses, initiating maternal tolerance induction to male transplantation proteins.^65–67^ This activation of adaptive immune responses against antigens in the seminal fluid may lead to the manifestation of autoimmune diseases, as well as AIBD, as sperm has been found to express E & N cadherins, which have interactions with Dsg, especially Dsg 2.^68,69^ If these molecules are foreign in sequence with the ones expressed in the female, they may initiate the production of autoantibodies that would damage the BM and reduce fertility (Table 2).

**Table 2:** Distinctive characteristics of the female gender regarding autoimmunity

Female predominance in autoimmune diseases (male:female = 10:1)^49–51^ and autoimmune bullous skin diseases^13^
The X-chromosome contains the largest number of immune-related genes of the whole human genome^52^ which play a major role in the onset, function and homeostasis of the immune system^53–56^
In the systemic circulation of females there are proteins which have slight variations and actions due to X-chromosome mosaicism. Autoreactive immune cells may not tolerate self-antigens encoded by one of the 2 X-chromosomes and therefore, they trigger autoimmune responses in target tissues^49,52^
Quality of life of female patients with autoimmune bullous skin diseases is affected more than men, due to time spent on treatment, social misconceptions, physical discomfort, and financial burden^57,58^
Pregnancy and lactation raise complicated management issues, including treatment limitations and a fluctuating disease course with postpartum exacerbations or relapses^13^
Hormonal changes during pregnancy may potentiate autoimmune disease activity, especially when the disease is uncontrolled at the time of fertilization^59^
During pregnancy, women are highly susceptible to autoimmune diseases, due to the necessarily immunotolerant state that occurs during childbearing^60^
Pemphigoid gestationis presents initial onset during pregnancy^13^
Pemphigus vulgaris improves after the third trimester of pregnancy and then flares postpartum^61^
For pregnant women with AIBD the delivery should be vaginal, as slow wound healing may occur after cesarean delivery, associated with corticosteroid use^62^
The activation of adaptive immune responses against antigens in the seminal fluid may lead to the manifestation of AIBD, as sperm expresses E & N cadherins, which interact with desmogleins.^68,69^ If these molecules are foreign in sequence to the wild type in the female, they may initiate the production of autoantibodies that would damage the BM
Female pemphigus patients were found to be significantly more likely to have anti-TPO autoantibodies than male patients^46^

AIBD, autoimmune bullous diseases; BM, basal membrane; TPO, thyroperoxidase.

## Pemphigoid and psoriasis: Most common blistering disease in women and its related autoimmune disease

The most representative group of AIBD in women is pemphigoid disease.^13^ BP is the most common pemphigoid subtype affecting 1.2 to 4.3/100,000 people annually.^70,71^ It predominantly affects women, typically after the sixth decade of life^13,16^; however, it is more active and severe among young people.^2^

Many studies have shown a significant association between BP and psoriasis. In a population-based retrospective cohort study including 3,924 patients with BP and 19,280 age-, sex-, and ethnicity-matched controls, patients with BP were 2.6-fold more likely to develop psoriasis compared to controls (Hazard ratio 2.60, 95% Confidence Interval (CI) 1.59-4.27) and the prevalence of preexisting psoriasis was higher in patients with BP than in controls (1.7 vs 1.1% respectively, *p* < .001).^30^ Furthermore, in the same study, a history of psoriasis was associated with a 50% increase in the risk of BP (Odds Ratio (OR) 1.53, 95% CI 1.17-2.02).^30^ Patients with the dual diagnosis of both autoimmune diseases were younger, had a higher prevalence of smoking and hypertension and were treated more frequently with prolonged systemic and topical corticosteroids when compared to patients with BP only.^30^ The increased burden of hypertension in these patients may reflect their higher exposure to systemic corticosteroids.^30^ Additionally, the bidirectional association between BP and psoriasis was more prominent in male than females.^30^ Similarly, in a meta-analysis encompassing data from 4,035 patients with BP and 19,215 controls, the pooled OR for psoriasis with BP was found to be 2.5 (95% CI 1.4-4.6) and the pooled prevalence of psoriasis was 1.8-fold higher among males with BP compared to their female counterparts.^72^ Likewise, in a case–control study of 287 BP patients and 1,373 matched controls, the prevalence of psoriasis was higher in BP patients than in controls (OR 4.4, 95% CI 2.2-8.9) and this association was significant among both sexes.^73^ In a Taiwanese population-based study of 3,485 patients with BP and 17,425 matched controls, psoriasis was significantly associated with BP (OR 2.02, 95% CI 1.54-2.66).^74^ Also, in a study of 145 case series of coexisting psoriasis and AIBD, BP was the most prevalent one (63.4%).^75^

The pathophysiological interpretation of this findings remains unknown, yet several hypotheses have been proposed. Notably, there are no common susceptibility human leukocyte antigen (HLA) alleles to overlap between these diseases and their common denominator is the BM.^30^ In psoriasis, the degradation of laminin 1 and laminin a1 in the BM is accelerated by the overexpression of fibronectin, α5β1 integrin, and plasminogen activators modifying the antigenicity of the BM and lowering the threshold for the generation of anti-BM autoantibodies that are also involved in BP.^24^ Another hypothesis mentions the shared role of neutrophils in both BP and psoriasis, as keratinocytes produce neutrophil chemoattractants and there is a neutrophilic infiltrate histologically present in both conditions.^25^ Neutrophils secrete several metalloproteases that may be implicated in the degradation of matrix proteins, leading to the exposure of BM antigenic epitopes.^26^ Moreover, interleukin (IL)-1 plays a central role in both psoriasis and BP, as it is essential for the initiation and formation of psoriatic lesions^27^ and is also increased in blister fluid of BP,^28,29^ correlating with the intensity of BP.^29^ Likewise, IL-17 and T helper type 17 cells play a major role in the pathogenesis of both BP and psoriasis.^30,31^

## Pemphigoid and neurological diseases

BP has been associated with a wide range of neurological diseases^76–81^ and patients with BP were found to be 5 times more likely to have any neurological disease, such as stroke (OR 1.85, 95% CI 1.55-2.19), Alzheimer’s disease (OR 2.11, 95% CI 1.73-2.57), Parkinson’s disease (OR 2.71, 95% CI 2.19-3.35), and epilepsy (OR 2.18, 95% CI 1.72-2.77).^36,82^ This association might be attributed to the co-expression of epithelial and neuronal isoforms of BP autoantigens (BP180 and BP230) in the skin and the central nervous system, respectively.^35^ That is to say, autoantibodies against the neuronal isoforms of BP180 and BP230 may lead to neuroinflammation and this may result in a cross-reactive immune response against their cutaneous isoforms, leading to BP.^36^

Additionally, patients with BP and comorbid neurological conditions were experiencing a more recalcitrant course of BP^83^ and the levels of BP autoantibodies were correlated with more severe dementia in Alzheimer’s disease.^84^ Moreover, patients with BP were more than 12 times likely to have multiple sclerosis.^82^

## Pemphigus and myasthenia gravis

Pemphigus can be associated with other autoimmune diseases in about 25% of patients.^85^ In some cases, it can be sporadic; however, for some associations, there is a common pathogenic mechanism.^86^

The pathophysiology of pemphigus and myasthenia gravis (MG) includes an autoantibody-mediated, non-cytotoxic mechanism, which can operate even in the absence of complement and inflammation.^86^ Approximately 95% of cases of MG are characterized by autoantibodies against the acetylocholine receptor (AChR), mainly nicotinic AChR (nAChR), and the muscle-specific kinase.^87,88^ Both pemphigus and muscle-specific kinase MG are mediated by antigen-specific autoantibodies of the IgG4 subclass^5,37^ and are associated with similar variants of the HLA class II region, such as DRB1*14, DQB1*05, and DRB1*14.(Figure 1) Autoantibodies against both muscarinic AChR and nAChR are also reported in pemphigus^39^ and that is because muscarinic AChR and nAChR are both expressed on the keratinocyte membrane and regulate cell adhesion synergistically.^40^

## Bullous diseases and autoimmune thyroid disease

The role of thyroid hormones in the development of the skin and its functions is well recognized, as thyroid hormones play an important role in fetal epidermal differentiation, hair growth, sebum production, wound healing, epidermal oxygen consumption, keratinocyte proliferation, and keratin gene expression.^12^

AITD are the most frequent autoimmune disorders with an estimated prevalence of 7 to 8%^14,46,89^ and a reported female predominance of 9:1.^46^ They include 2 main clinical entities: (a) chronic lymphocytic thyroiditis (or Hashimoto thyroiditis), which leads to hypothyroidism and (b) Graves’ disease (or Basedow disease), leading to hyperthyroidism.^12,14,89^ Hashimoto thyroiditis is characterized by the production of anti-thyroglobulin (anti-Tg) and anti-thyroperoxidase (anti-TPO) autoantibodies, which progressively lead to gland fibrosis and hypothyroidism. In contrast, Graves’ disease is portrayed by the production of stimulating autoantibodies against the thyrotropin receptor that cause unrestricted thyroid hormone synthesis and release.^90^

BP has been associated with both Hashimoto thyroiditis and Graves’ disease.^91–93^ Indeed, there is a higher prevalence of anti-TPO antibodies in BP when compared to control subjects and this was not observed in pemphigus patients.^94^ Women affected by gestational pemphigoid demonstrated a higher prevalence of Hashimoto thyroiditis and Graves’ disease.^16^

DH is associated with AITD (mainly Hashimoto thyroiditis) and up to 48% of DH patients were found positive for anti-TPO autoantibodies in several studies.^15,16,95,96^

AITD is the most commonly self-reported autoimmune disease in PV patients and/or their first-degree relatives.^97,98^ Several studies reported a significantly higher prevalence of anti-TPO and anti-Tg autoantibodies in pemphigus patients compared to controls.^12,46,99–101^ Yet, anti-TPO and anti-Tg autoantibodies are not different between patients with active PV and patients with remittent disease, indicating that these antibodies are related to disease expression and not disease activity.^46^ Moreover, patients with cutaneous only lesions have a significantly higher prevalence of anti-Tg autoantibodies than those with mucosal or mucocutaneous lesions.^46^ Additionally, in PV there is a significant association between HLA type and anti-TPO antibody levels. Many studies have established a strong association between DRB1*04:02 and DQB1*05:03 and increased risk for PV.^46,102–104^ Yet DRB1*04:02-/DQB1*05:03-patients have the highest prevalence of anti-TPO.^46^ Indeed, the absence of DQB1*05:03 (regardless of the presence of DRB1*04:02) predisposes a patient with PV to develop anti-TPO antibodies.^46^ Furthermore, in PV, the absence of both anti-Dsg 1 and anti-Dsg 3 autoantibodies has been correlated with highest anti-thyroid activity (increased anti-TPO and anti-Tg), followed by the absence of anti-Dsg 1 alone.^46^ Also, Dsg1-/Dsg3-PV patients in active disease showed significantly higher levels of anti-Tg than Dsg1-/Dsg3-PV patients in remission.^46^ Interestingly, female pemphigus patients were found to be significantly more likely to have anti-TPO autoantibodies than male patients.^46^

The above-mentioned association of bullous skin diseases with AITD can be attributed to the fact that different skin cell types, including keratinocytes, melanocytes, and dermal fibroblasts bear functional thyrotropin receptor and other thyroid-specific antigens, such as Tg, TPO, and natrium/iodide symporter.^41–45^

## EBA and Crohn’s disease

Inflammatory bowel disease (IBD) is characterized by aggressive, cytokine-driven, non-infectious inflammation of the gut, caused by T-cells and antigen presenting cells producing pro-inflammatory cytokines (IL-6, Tumor Necrosis Factor-α), which cause mucosal inflammation and destruction.^47,105^ Cutaneous symptoms in IBD occur with an incidence of up to 40% and the most common are erythema nodosum and pyoderma gangrenosum.^106,107^

EBA has been associated with various systemic diseases^21,108^; however, the strongest association was found with IBD and mainly Crohn’s disease.^12^ About 68% of IBD patients have circulating autoantibodies against C7 and Crohn’s disease has been detected in about 30% of EBA patients.^21,109^

The possible pathological mechanisms explaining the association of EBA with IBD could be the expression of C7 in both colon and skin, which leads to a cross-reaction to the same antigen in different tissues.^12^ Thus, in EBA patients, C7 could harbor the primary epitope of a spreading phenomenon toward IBD autoimmunity.^21^ However, IBD usually precedes or occurs simultaneously with the onset of blistering.^47^ It is also possible that C7 expressed in the mucosa of the colon is altered by chronic inflammation of IBD and thus cryptic epitopes of the protein are revealed, or neo-epitopes are generated.^47,48^ An alternative mechanism could be related to molecular mimicry: the immune response directed against pathogens, or the intestinal flora could induce the initial activation of T and B cells, which are cross-reactive with C7 epitopes, provoking the generation of autoantibodies that trigger blister formation in the skin.^47^ The autoantibodies against C7 in EBA and IBD belong to different subclasses: EBA is characterized mainly by IgG1 and IgG4 subclasses, whereas IBD is characterized by the IgG3 subclass and this might explain the absence of skin blistering in the majority of patients with IBD.^47,110^

Ulcerative colitis has also been associated with autoantibodies against C7 and EBA, however this association is less frequent than the association with Crohn’s disease.^48,111^ This might be attributed to the fact that there is a higher incidence of C7 autoimmunity in Crohn’s disease.^47^

## Conclusions

AIBD have a significant female predominance and present many associations with other severe autoimmune diseases. Clinicians should be aware of these associations, especially when treating women with bullous diseases, in order to be able to aid them with serious decisions regarding pregnancy and lactation planning. Moreover, the continuous investigation of these associations in the future may shed light to important molecular and immunological pathogenic mechanisms of bullous skin disorders that remain unknown.

## Conflicts of interest

None.

## Funding

None.

## Study approval

N/A.

## Author contributions

MK, DFM, AK, AP: Literature research, writing of the manuscript, contribution to the finalization of the manuscript and approval of the submitted article. AP: Conception of the research project, contribution in integrity and accuracy of data, preparation and approval of the submitted article.
